# Effect of Infla-Kine supplementation on the gene expression of inflammatory markers in peripheral mononuclear cells and on C-reactive protein in blood

**DOI:** 10.1186/s12967-017-1315-4

**Published:** 2017-10-20

**Authors:** Nina A. Mikirova, Santosh Kesari, Thomas E. Ichim, Neil H. Riordan

**Affiliations:** 1 The Riordan Clinic, Wichita, KS USA; 2 Department of Translational Neuro-Oncology and Neuro-therapeutics, John Wayne Cancer Institute at Providence Saint John’s Health Center, Santa Monica, CA USA; 3 Immune Advisors LLC, San Diego, CA USA; 4 Aidan Products, Dallas, TX USA

## Abstract

**Background:**

Chronic inflammation is a predisposing factor to numerous degenerative diseases including cancer, heart failure and Alzheimer’s disease. Infla-Kine is a natural supplement comprised of a proprietary blend of *Lactobacillus fermentum* extract, burdock seed (arctigenin), zinc, alpha lipoic acid, papaya enzyme and an enhanced absorption bio-curcumin complex (BCM-95^®^).

**Methods:**

Infla-Kine was administered twice daily to 24 health volunteers for 4 weeks. Quantitative RT-PCR was used to assess mRNA transcripts of IL-1b, IL8, IL-6, NF-κB, and TNF-α from peripheral blood mononuclear cells (PBMC). C reactive protein (CRP) was measured from serum. Additionally, quality of life questionnaires were employed to assess general feeling of well-being. Assessments were made before treatment and at conclusion of treatment (4 weeks).

**Results:**

As compared to pre-treatment, after 4 weeks, a statistically significant reduction of IL8, IL-6, NF-κB, and TNF-α transcripts was observed in PBMC. Furthermore, reduction of IL-1b transcript and serum CRP was observed but did not reach statistical significance. Quality of life improvements were most prevalent in muscle and joint pains.

**Conclusions:**

Overall, our data demonstrate that twice daily administration of Infla-Kine for 4 weeks reduces inflammatory markers and quality of life in healthy volunteers.

## Background

Chronic inflammation is associated with persistent, yet low grade, activation of various bodily defense factors which often results in deleterious effects to the host. In a healthy immune response inflammatory mediators, such as cytokines, are upregulated in response to a pathogen. Subsequent to clearance of the pathogens the factors are downregulated. In contrast, in chronic inflammation the same molecular mediators that are used to protect the host against pathogens often become the cause of pathology due to their chronic production. For example, TNF-alpha is a cytokine produced by innate immune cells such as macrophages and is involved in host responses to various bacteria such as tuberculosis. During the natural course of an immune response, subsequent to clearing of mycobacterium tuberculosis, production of TNF-alpha subsides. In contrast, in conditions such as obesity, excess adipocytes directly secrete TNF-alpha [1], as well as produce agents that induce production of TNF-alpha such as HMGB-1 [2, 3]. Although originally correlative studies existed between obesity and comorbidities such as Type 2 diabetes and cardiovascular diseases, the era of cellular and molecular medicine has led to specific causal effects of chronic inflammation in evolution of pathologies. In the example of Type 2 diabetes, TNF-alpha have been shown to be directly associated with induction of insulin resistance, in part through suppression of the insulin receptor substrate component of insulin receptor signaling [4–7]. In the example of cardiovascular disease, inflammatory mediators, such as, TNF-alpha [8, 9], IL-1 beta [10], and IL-6 [11], have been shown to be directly inhibitory to endothelial progenitor cells (EPC). EPC are known to play a fundamental role in regenerating vascular endothelium and EPC levels negatively correlate with cardiovascular risk factors [12, 13].

The prospect of a natural supplement reducing chronic inflammation has potential in the treatment of numerous conditions in which inflammation plays a major pathogenic role. In addition conditions classically associated with chronic inflammation such as diabetes and cardiovascular disease, chronic inflammation has been shown to be important in other conditions. For example, in cancer patients, chronic inflammatory conditions are associated with stimulation of pathological angiogenesis [14–17], as well as immune suppression [18]. In dialysis patients, inflammatory mediators are believed to play a role in the overall shorter survival of patients [19–23]. Even psychiatric conditions such as panic disorder have been reported to possess an inflammatory element [24, 25].

Infla-Kine is a commercially available nutraceutical supplement comprised of a proprietary blend of *Lactobacillus fermentum* (LF) extract, burdock seed (arctigenin), zinc, alpha lipoic acid, papaya enzyme and an enhanced absorption bio-curcumin complex (BCM-95^®^). *Lactobacillus fermentum* extract has proven health benefits [26]. It is natural immune booster and has been shown to trigger a cascade of events that help regulate the immune system, making it stronger and more efficient. In addition, the administration of LF was associated with significant mobilization of cells expressing hematopoietic stem cell markers [27], which is extremely important for health, as numerous studies demonstrate a direct, positive correlation between the number of circulating stem cells and health, wellness and regenerative capabilities [28].

The current study assessed whether levels of inflammatory mediators were affected by administration of Infla-Kine for 4 weeks in healthy volunteers. The results support the use of this natural-based food supplement for modulation of chronic inflammatory states, and thus potentially reducing a variety of health risks.

## Methods

### Recruitment of subjects

Twenty-four subjects (20 women and 4 men) were recruited for a short-term (4 weeks) study in order to assess the effect of Infla-Kine supplementation on gene expression of inflammatory cytokines in PBMCs and serum C-reactive protein. Subjects were chosen among the employees of the Riordan Clinic and provided written informed consent to participate in the study. The research was in compliance of the declaration of Helsinki and approved by the Institutional Review Board of Riordan Clinic.

All participants were in good health as determined by a medical history and clinical laboratory tests. Subjects fulfilled the following criteria:

1. No history of chronic disease.
2. No antibiotic use for 2 weeks before the beginning of the study.
3. Nonsmoking.
4. No drugs or nonsteroidal inflammatory drugs 2 weeks before and during the study.

Subjects with Type 1 diabetes mellitus, autoimmune diseases, malignant diseases, and infectious diseases were excluded from the study. Participants maintained their usual habits including physical, sleeping habits and diet during the study.

After enrollment, participants took at least 2 capsules of supplement Infla-Kine daily for month. Blood samples were drawn at the beginning of the study and at the end of the study.

### Isolation of PBMC

Whole blood was collected by venipuncture into heparinized tubes. For PBMC collection, blood was diluted 1:1 with phosphate buffered saline (PBS), layered on top of Ficoll-Paque Plus (Amersham Biosciences), and centrifuged at 400*g* for 30 min at 4 °C. PBMC were then removed from the plasma-Ficoll interface and rinsed twice with PBS.

CRP in blood serum (collected by venipuncture and centrifugation) was measured by a licensed and certified medical Bio-Center Laboratory of the Riordan Clinic (CLIA 17D0648333) by standard procedure.

### RNA extraction and qRT-PCR

After separation of PBMCs, 1 mL of TriReagent (Sigma-Aldrich, Hercules CA) was added for RNA extraction following manufacturer’s instructions. Total RNA quality and quantity was evaluated using the Nanodrop ND-2000 (Thermo Scientific, Pittsburg PA) and subsequently converted to cDNA using the iScript RT supermix in the CFX96 Real-Time PCR Detection System (Bio-Rad, Hercules, CA, USA). cDNA was than quantified using the Nanodrop ND-2000 and a total of 250 ng were used to analyze gene-specific oligonucleotide primers (Table 1) with the SsoAdv Universal SYBR GREEN Kit. A dissociation curve was run at the end of the reaction to ensure that only one amplicon was formed and that the amplicons denatured consistently in the same temperature range for every sample. The cDNA levels were normalized against housekeeping gene ribosomal protein 13 (RSP13).

**Table 1:** Oligonucleotide primers and PCR conditions for inflammatory response genes

Genbank access. #	Symbol and description	Primers	qPCR^a^ (°C)
NM_000576.2	IL1β Interleukin 1 beta	HsIL1BF: ggagaatgacctgagcacct HsIL1BR: ggaggtggagagctttcagt	56
NM_000584.3	CXCL8 Interleukin 8	HsIL8F: cagttttgccaaggagtgct HsIL8R: acttctccacaaccctctgc	58
NM_000594.3	TNF Tumor necrosis factor	HsTNFF: gtcaacctcctctctgccat HsTNFR: ccaaagtagacctgcccaga	57
NM_001165412	NFκB Nuclear factor kappa B	NFκBF: gcacgacaacatctcattgg NFκBR: tcccaagagtcatccaggtc	58
NM_001017.2	RSP13 Ribosomal protein 13	RPS13F: cgaaagcatcttgagaggaaca RPS13R: tcgagccaaacggtgaatc	57
NM_000600.3	IL6 Interlukin 6	HsIL6F: agtcctgatccagttcctgc HsIL6R: aagctgcgcagaatgagatg	56

^a^Initial denaturation at 98 °C for 30 s, followed by forty cycles of denaturation at 95 °C for 10 s, annealing for 15 s (at temperature given) and extension at 60 °C for 15 s

### Health questionnaire

A multi-item scale based questionnaire was used which included symptoms (fever, chills, headaches, loss of appetite, joint pain, joint stiffness, constipation, diarrhea, bloating), a global health improvement (overall improvement of physical conditions) and overall improvement of the quality of life. The range in score was from 0 to 4 for symptoms and from 1 to 7 for overall improvement.

Signs and symptoms were allocated intensity scores of 0 (never or almost never have the symptom); 1 (occasionally have it, mild symptoms); 2 (occasionally have it, severe symptoms); 3 (frequent have it, mild symptoms); 4 (frequently have it, severe symptoms). The overall improvement of the physical conditions and quality of life was rated with a difference of 1–7 points, 1 representing a small change and 7 representing the largest improvement.

In efficacy analysis, the symptom intensity scores were considered as quantitative variables and values were calculated as the percentage of the participants who had the symptom with certain score to total number of participants.

### Statistical analysis

The analysis and comparisons of mRNA expression levels were carried out using the Kaleidagraph (Synergy Software, Reading PA, USA) and Systat Software (San Jose, CA, USA) statistical software. Data are presented as mean ± SD. Pre and post treatment comparison was performed using paired t tests and ANOVA. Differences in mean values were considered significant at the level of 95% (p < 0.05). Outliers in gene expression data were removed based on the interquartile range test. The 2^−ΔΔCt^ method was used to calculate differences in gene expression.

## Results

### Baseline demographics

The sample size was 24 subjects (4 men and 20 women) without any systemic diseases. The average age of participants was 48.4 ± 14.7 years old. There was trend towards adiposity in group of participants (higher than normal range of BMI 30.9 ± 8.1 and elevated cholesterol 210.0 ± 56.4 mg/dL at normal level < 200 mg/dL). Most of the participants had inflammation and increased level of CRP. In average CRP was 3.95 ± 3.85 mg/L at normal level < 2 mg/L (interquartile range IQR 1.1–6.9 mg/L). Eight subject had CRP three times higher than normal range and the maximum level of CRP reached 13.8 mg/dL.

### Effect of supplementation on gene expression of inflammatory markers

**Fig. 1:**
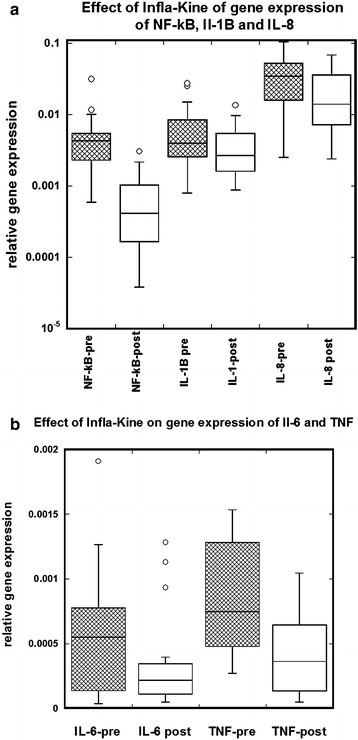
Effects of 4 week Infla-Kine supplementation on PBMC inflammatory gene expression. Gene expression was assessed by RT-PCR as indicated in materials and methods from patient PBMC prior to initiation of Infla-Kine administration (Pre) and 4 weeks after (Post). Relative gene expression was quantified based on housekeeping gene control. **a** Depicts NF-kappa B, IL-1 beta and IL-8 expression, whereas **b** depicts IL-6 and TNF-alpha expression

Blood samples were extracted, and levels of mRNA expression from isolated peripheral blood mononuclear cells (PBMC), from healthy volunteers before and after administration of Infla-Kine for 4 weeks. As shown in Fig. 1 and Table 2, PBMC mRNA expression levels of various cytokines during the time course of the study. Data in Table 2 demonstrate the average with SD of the expressions of IL-1b, IL8, IL-6, NF-κB, and TNF-α before and after intervention. Outliers in gene expression data were removed based on the interquartile range test. The last column in Table 2 shows the p values calculated by paired t test. It was observed that IL-8, IL-6, TNF-α and NF-κB showed statistically significant changes after 4 week administration of Infla-Kine. For the cytokine IL-1b gene expression level decreased, but did not reach statistical significance (p < 0.13). According to our data, the gene expression of inflammatory cytokines was downregulated 1.6 times for IL-6, 1.4 times for TNF-α, 2.0 times for IL-8 and 1.4 times for IL-1b. The gene expression of transcriptional factor NF-κB was downregulated 5.6 times as the result of intervention.

**Table 2:** PBMC mRNA expression levels of various cytokines and transcriptional factors before and after intervention

	Before		After		p value (one tail)
	Average	SD	Average	SD	
NF-κB	0.00388	0.00237	0.00068	0.00074	0.0001
IL1B	0.00483	0.00388	0.00348	0.00239	0.13
IL8	0.03506	0.02595	0.01735	0.01578	0.003
TNF-α	0.00097	0.00088	0.00068	0.00112	0.004
IL-6	0.00057	0.00053	0.00036	0.00037	0.006

### Effect of Infla-Kine supplementation on CRP

In addition to the gene expression of pro-inflammatory cytokines in PBMCs, we measured the levels of C-reactive protein (CRP), an acute phase protein, which is a sensitive systemic marker of inflammation and acute-phase reactions. CRP values may increase by several folds by de novo hepatic synthesis regulated by pro-inflammatory cytokines, especially interleukin-6 [29]. A major CRP response is observed in infection and sepsis, various auto-immunopathies, tissue necrosis, trauma, and neoplasia [30].

**Fig. 2:**
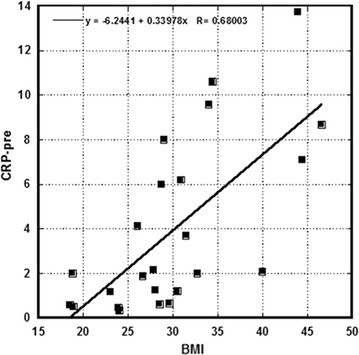
Correlation of CRP and body mass index prior to intervention. CRP and BMI were quantified as described in materials and methods. A positive correlation was observed between CRP and BMI

Data presented in Table 3 show the measured CRP levels before and after intervention and the body mass indexes (BMI) of participants. The subjects recruited in this study tended toward obesity, as normal BMI is in range 18.5–24.9; for overweight subjects the range is 25–29.9 and in obese subjects 30.0–34.9. Increased level of BMI showed correlation (r = 0.7) with the levels of the inflammation marker CRP (Fig. 2). The average values of CRP before after supplementation with p values (paired t test, one-tail) are presented in Table 4. The data are shown for all subjects and for subjects with initial CRP higher than normal range (CRP > 2 ng/mL). The average values of CRP for both groups were reduced, but the difference did not reach statistical significance (p values 0.17 and 0.19).

**Table 3:** Patient level CRP data

Subjects	BMI	CRP-pre	CRP-post	Subjects	BMI	CRP-pre	CRP-post
1	30.9	6.2	8.74	13	26.6	1.89	1.61
2	23.9	0.48	0.37	14	28	1.27	1.45
3	32.7	2	1.64	15	28.5	0.62	0.55
4	27.8	2.18	2.98	16	18.9	0.53	0.58
5	43.9	13.76	18.5	17	24	0.33	0.33
6	44.4	7.12	3.74	18	23	1.18	1.1
7	29.6	0.67	0.91	19	28.7	6.01	5.95
8	18.5	0.6	0.16	20	34	9.58	6.86
9	31.4	3.7	2.42	21	29	8.01	4.09
10	18.8	2	1.5	22	26	4.13	2.52
11	40	2.09	1.96	23	30.5	1.2	0.48
12	46.6	8.67	5.77	24	34.4	10.6	11.35

**Table 4:** Summary of CRP levels before and after treatments

	CRP	Pre	Post	p value
All subjects	Average	4.04	3.65	0.17
	SD	3.91	4.38	
Subjects with CRP > 2 ng/mL	Average	6.14	5.58	0.19
	SD	3.70	4.71	

**Fig. 3:**
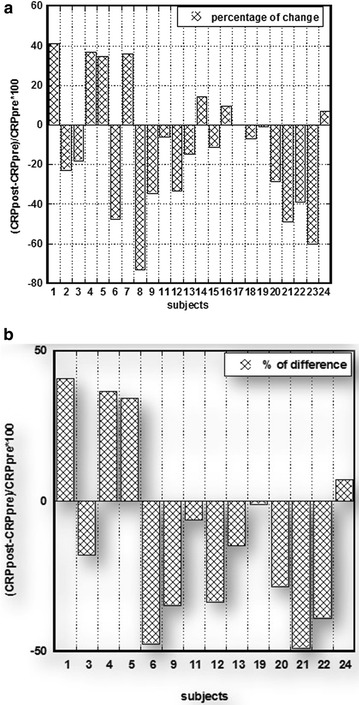
Modification of CRP levels by 4 week Infla-Kine supplementation. The percentage of difference in CRP measured pre and post intervention is shown for all subjects in **a** and for subjects with abnormal initial levels of CRP in **b**. Negative values demonstrate the improvement in the CRP concentrations after intervention and positive values—increase in these values

The percentage of difference in CRP measured pre and post intervention is shown for all subjects in Fig. 3a and for subjects with abnormal initial levels of CRP in Fig. 3b. Negative values demonstrate the improvement in the CRP concentrations after intervention and positive values—increase in these values. According to our data, treatment during 1 month by oral supplement Infla-Kine was associated with a decrease in CRP levels in 62% of all participants and in 71.4% of participants with CRP higher than upper normal level (CRP > 2 ng/mL).

### Improvement in health questionnaire

The calculated values are presented in Table 5 and showed the percentage of participants who had any of the listed symptoms sorted according to the score (0–4) and the percentage of participants. Subjects who were on Infla-Kine supplementation also declared at the end of the study the improvement of the physical conditions and quality of life on scale from 1 (poor) to 7 (very good). 70% of patients declared improvement in physical conditions and the quality of life at the level higher than four. The highest level of improvement 15–20% was in conditions of muscle and joint stiffness and joint pain. 10% indicated the improvement in the energy level and constipation. Only 5% of the subjects had complaints of worsening conditions.

**Table 5:** Changes in health questionnaire

Symptom scale	Percentage of participants having symptoms before intervention (0-never, 1-occasionally, 2-occasionally severe, 3-frequent, 4-frequent severe)					Changes in the health status after intervention		
	0	1	2	3	4	Same	Better	Worse
Fever	95	5	0	0	0	100	0	0
Chills	95	0	0	5	0	100	0	0
Fatigue	50	25	10	10	5	85	10	5
Headaches	75	10	10	5	0	95	5	0
Muscle stiffness	55	25	5	15	0	80	15	5
Loss of appetite	90	0	10	0	0	90	5	5
Joint pain	75	10	5	10	0	85	15	0
Joint stiffness	75	10	10	5	0	80	20	0
Constipation	80	10	5	5	0	85	10	5
Diarrhea	80	20	0	0	0	95	5	0
Bloating	80	10	10	0	0	90	5	5

## Discussion

The effects of the Infla-Kine supplementation on gene expression and transcriptional factor modulation in peripheral blood mononuclear cells from 24 subjects were determined. Participants received two capsules per day for 4 weeks. The expression profile of several genes related to the inflammation was quantified by real time RT-PCR. In addition, C-reactive protein was measured at the beginning and the end of intervention.

Many subjects in this study tended toward obesity, and showed typical symptoms of adiposity, including abnormally high levels of the inflammation marker CRP. Our data demonstrated a correlation between CRP and BMI (r = 0.7) before intervention. This is consistent with observations in the literature that inflammation levels are increased in overweight and obese subjects [31, 32]. According to the other studies, conditions associated with adiposity are also accompanied by changes in PBMC gene expression favoring inflammation [33]. Pro-inflammatory cytokines such TNF-α and IL-6 are overexpressed in obese subjects, as is nuclear factor kappa-light-chain-enhancer of activated B cells (NF-κB), the transcription factor that controls many genes associated with inflammation [34]. Moreover, subjects with adiposity have reduced serum levels of the anti-inflammatory cytokines [35].

According to our study, gene expression of pro-inflammatory cytokines was significantly affected by Infla-Kine supplementation. IL-8, IL-6, and TNF-α showed statistically significant downregulation during the time frame of the study. For the cytokine IL-1b the gene expression levels was decreased, but did not reach statistical significance. The supplementation caused downregulation of transcriptional factor NF-κB (p < 0.0001). NF-κB is considered a prototypical pro-inflammatory signaling pathway, largely based on the activation of NF-κB by pro-inflammatory cytokines such as IL-1 and TNF-a, and the role of NF-κB in the expression of other pro-inflammatory genes including cytokines, chemokines, and adhesion molecules, which has been extensively reviewed elsewhere [36].

Another measured parameter was CRP, which directly correlates with disease activity in many diseases and can contribute to disease progression through a range of pro-inflammatory properties. Being an exquisitely sensitive marker of systemic inflammation and tissue damage, CRP is very useful in screening for organic disease, monitoring treatment responses, and detecting infection.

Our data demonstrated that the intervention by supplementation showed positive effect on the concentrations of CRP. The treatment by oral supplement Infla-Kine during 1 month was associated with a decrease in CRP levels in 62% of all participants and in 71.4% of participants with CRP higher than upper normal level (CRP > 2 ng/mL).

At the end of the study each participant filled the questionnaire, which included symptoms (fever, chills, fatigue, headaches, loss of appetite, joint pain, joint stiffness, constipation, diarrhea, bloating), a global health improvement (overall improvement of physical conditions) and overall improvement of the quality of life. 70% of patients declared improvement in physical conditions and the quality of life at the level higher than four on scale from 1 to 7. The highest level of improvement was in conditions of muscle and joint stiffness and joint pain.

## Conclusion

Infla-Kine, a commercially-available natural supplement, reduced IL-8, IL-6, NF-κB, and TNF-α mRNA transcripts in PBMC of 24 healthy volunteers after twice daily administration for 4 weeks. Reduction of CRP and IL-1b transcripts was also observed but did not reach statistical significance. Subjects reported improvements in muscle and joint pains in quality of life questionnaire. Overall these data support the use of Infla-Kine supplementation as a means of decreasing chronic inflammation in otherwise healthy subjects.
